# Walkable Dual Emissions

**DOI:** 10.1038/srep02199

**Published:** 2013-07-16

**Authors:** Hai-Bing Xu, Peng-Chong Jiao, Bin Kang, Jian-Guo Deng, Yan Zhang

**Affiliations:** 1 New Materials R&D Center, Key Laboratory of Science and Technology on High Energy Laser, Institute of Chemical Materials, China Academy of Engineering Physics, Chengdu, Sichuan 610200, China; 2 State Key Laboratory of Structural Chemistry, Fujian Institute of Research on the Structure of Matter, Chinese Academy of Sciences, Fuzhou, Fujian 350002, China

## Abstract

Walkable dual emissions, in which the emission bands of the walker reversibly cross or leave those of the stationary ones depending on temperature and concentration, have been demonstrated in cyclic dimeric lanthanide complexes [Ln(hfac)_3_(PAnPO_2_)]_2_ (Ln = Eu^III^, Tb^III^; hfac^−^ = hexafluoroacetylacetonate; PAnPO_2_ = 9,10-bis(diphenylphosphino) anthracene dioxide), providing a concept model for signals exchanging and dispatching. Additionally, good linear relationships are observed between the maximum emission bands of the walker *vs* the concentrations {lg(*M*)} and the measurement temperatures (*K*), implying such materials could be served as potential concentration and temperature sensors.

Molecular information process, operative in molecular system with reversibly switchable states, is a hot topic^1^,^2^. These processes are commonly controlled by stimuli, such as by chemical^3^ or electrical^4^ energies, as well as by light^5^. Like bead shuttling back and forth along the thread in rotaxane^6^, one emission bands reversibly crossing or leaving the static ones in single molecule depending on experimental conditions named as “Walkable Dual Emissions” (WDE), may be potential application in signal transmission.

We consider that a model with a typical “WDE” should match the following two criteria: 1) There are at least two types of luminophores in a molecule, one is the stationary, the emission bands of which are not affected by peripheral environment, such as the lanthanide subunits in its complex^7^. While the other is the walker, the emission behavior of the walker, such as the aromatic ligands, is sensitive to the external environment^8^. 2) The two chromophores are assembled in a cyclic structure. Given the two prerequisite conditions are warranted, the parallel conjugate aromatic ligands (the walkers) can easily suffer from *π*···*π* stacking interactions^8^,^9^, inducing the emission bands of the walker moving toward or backward those of the lanthanide ones (the stationary). When the emissions (signals) meet with each other, a possibility to exchange information is present. If the walker leaves the stationary, dispatching information becomes possible.

Herein, we report a cyclic dimeric lanthanide [Ln(hfac)_3_(PAnPO_2_)]_2_ (**1**, Ln = Eu^III^, Tb^III^; hfac^−^ = hexafluoroacetylacetonate; PAnPO_2_ = 9,10-bis(diphenylphosphino) anthracene dioxide) that exhibits two emission bands, in which emission bands from PAnPO_2_-based luminophore reversibly cross or leave the fixed Ln^III^-based emissions depending on temperature and concentration. Emission from the lanthanide remains static throughout and serves as the stationary band to compare PAnPO_2_-based emission migration. The highly luminescent complex **1**·Eu was characterized by X-ray crystallography (CCDC 902262), IR, elemental analysis, ESI-MS (Figure S1, ESI), and photophysical properties.

## Results

### Synthesis and structure

**Figure 1:**
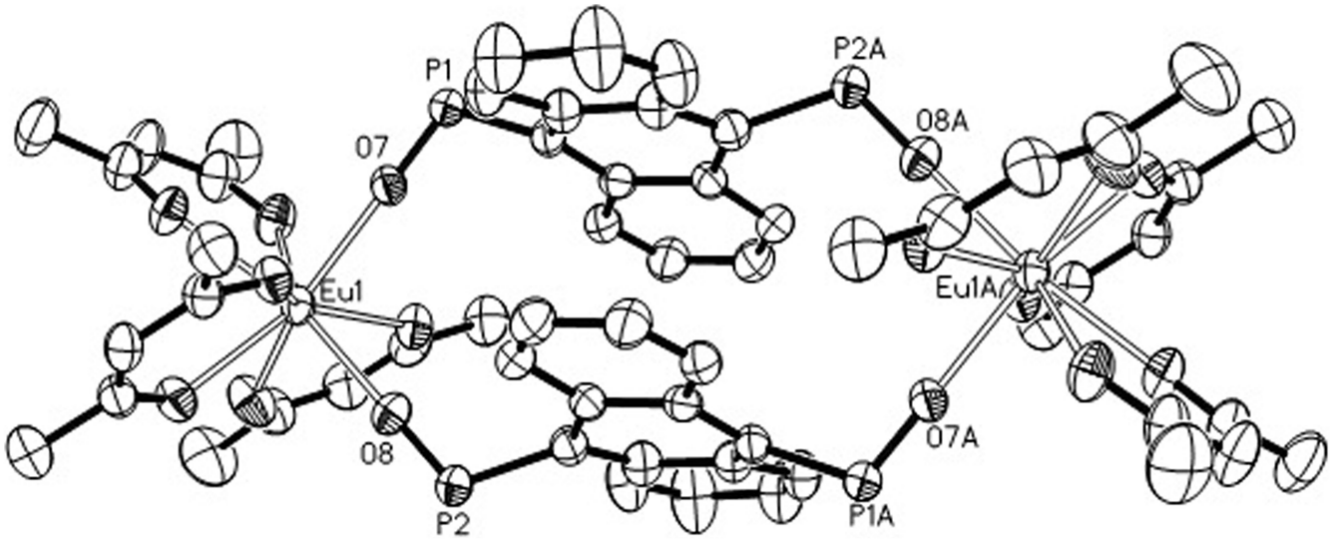
ORTEP drawing of **1**·Eu with atom labeling scheme showing 30% thermal ellipsoid. The solvent molecules, phenyl groups on phosphineoxide, as well as the H and F atoms on the hfac^−^ are omitted for clarity.

The cyclic lanthanide complexes could be afforded by two methods, one is *in situ* oxidation PAnP into PAnPO_2_ by O_2_, the other one is by prior addition of H_2_O_2_, then reaction of equal molar ratio of PAnPO_2_ with Ln(hfac)_3_(H_2_O)_2_ affords orange crystals of **1**. We found that the former method is better than the latter in the yields. As depicted in Figure 1, the crystal structure of **1**·Eu can be viewed as two Eu(hfac)_3_ subunits bridged by PAnPO_2_ into a cyclic structure. The Eu^III^ ion is encapsulated by eight oxygen atoms including six from hfac^−^, and two from the P = O groups to furnish a distorted square-antiprism. The bridging ligand PAnPO_2_ exhibits a “W” arrangement with a linear P-O-M angle (176.5(3)° and 178.0(3)°), comparable to those found in the phosphine oxide lanthanide complexes^10^, linking two Eu(hfac)_3_ units into a symmetric fashion with an Eu···Eu distance of ca. 11.395 Å. It is worthy to note that the two coplanar anthracenes in **1**·Eu experience strong *π*···*π* stacking interactions with the distance about 3.4 Å. Due to steric requirement, the two Eu(hfac)_3_ are oriented at opposite directions.

### Photophysical properties

The UV-vis absorption spectra of **1**·Eu and PAnPO_2_ (Figure S2, ESI) display moderately intense vibronic bands with similar band shapes around 350–500 nm, and the vibronic spacing in **1**·Eu (1422 and 2060 cm^−1^) is, being close to, yet red-shifted ca. 25 nm from those of the free PAnPO_2_ (1375 and 1616 cm^−1^), assigned as the low-energy ^1^*π* → π* transition of the anthracenyl rings^11^. Noticeably, the vibronic bands are extended from 450 nm in the free PAnPO_2_ to 500 nm in the cyclic **1**·Eu owing to the two coplanar anthracenes.

**Figure 2:**
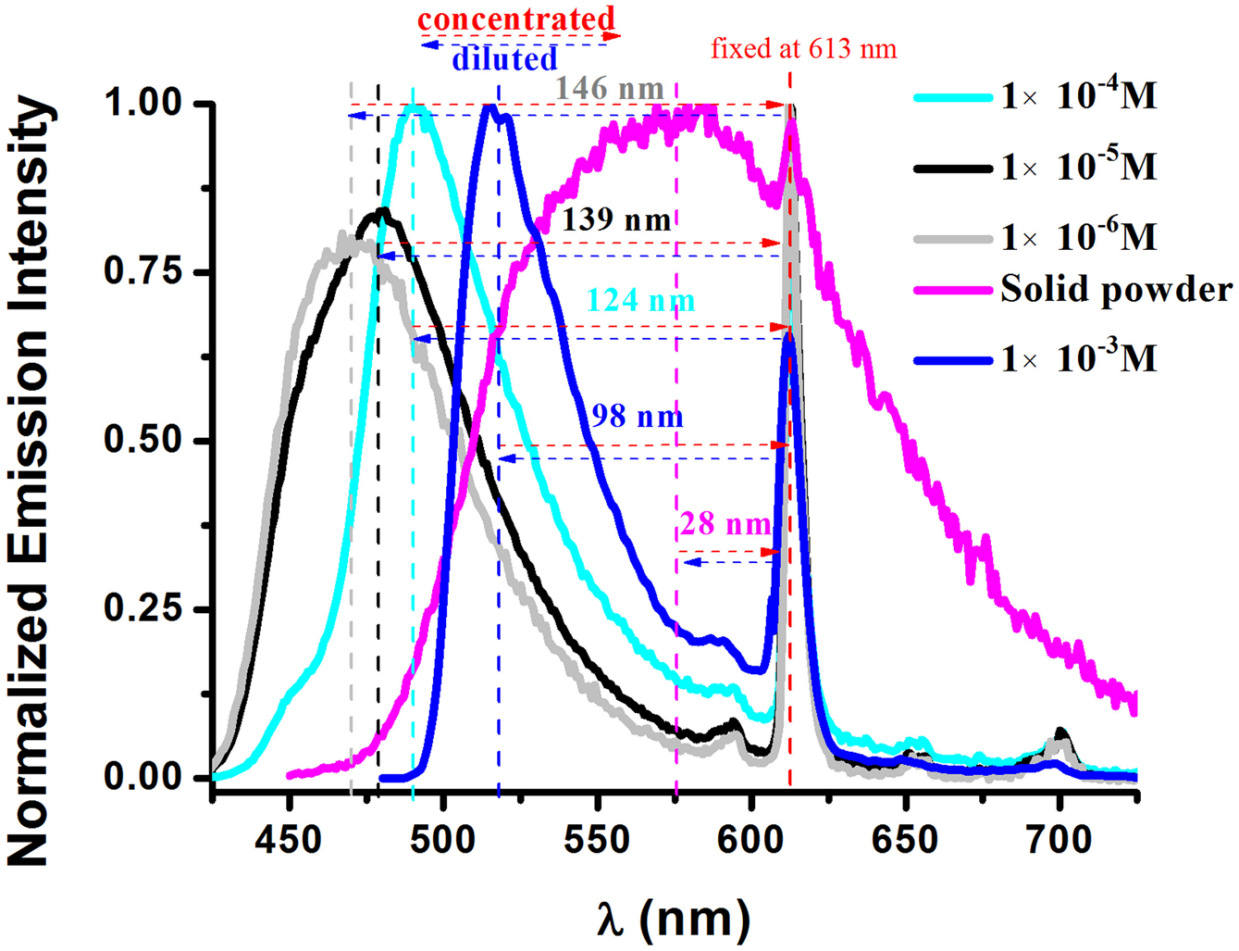
Normalized emission spectra of **1**·Eu (*λ*_ex_ = 350 nm) with increasing concentrations in dichloromethane solution at ambient atmosphere, showing the emission bands of the walker (PAnPO_2_) toward those of the stationary (Eu^III^-emitter).

Upon excitation with *λ*_ex_ = 384 nm, PAnPO_2_ exhibits a broad emission band centered at ca. 470 nm in an aerated dichloromethane solution at 298 K, ascribed to the low energy ^1^*π* → π* transitions in the anthracenyl ring^11^. Upon illumination at 350 nm, **1**·Eu (10^−5^ M) exhibits a broad luminescence band centered at ca. 475 nm as well as a narrow line-like emission at 613 nm, featured distinctly from PAnPO_2_ chromophore and Eu^III^ center, respectively (Figure 2).

### White-light emission

It has been demonstrated that white-light emission is accessible by mixing pure red, green, and blue emissions^14^ or at least two complementary color emissions^15^. As **1**·Eu (10^−5^ M) exhibits complementary dual emissions from both PAnPO_2_-based cyan emission at 475 nm and the sensitized Eu^III^-centred red emission at ca. 613 nm, this is very suitable for generating white-light emission^16^. The quantum yield of the white-light emission from **1**·Eu is 7.8% and the chromaticity coordinate CIE is (0.36, 0.35) by excited at 350 nm, within the white region of the International Commission on Illumination (CIE) 1931 color space chromaticity diagram (Figure S5, ESI)^17^.

### Energy transfer procedure

Due to the internal heavy atom effect, and lacking of energy transfer from the sensitizer triplet states, **1**·Gd is a suitable candidate for evaluating ligand triplet energy levels, so as to assess its efficiency in the energy-transfer process to the lanthanides. As shown in Figure S3, **1**·Gd exhibits ligand phosphorescence as a broad band centered at ca. 472 nm with vibrational structure after excitation at 380 nm in degassed methanol at 77 K. The calculated triplet-state energy level (21300 cm^−1^) is favorable for energy transfer into those of Eu^3+^ (^5^D_0_, 17300 cm^−1^), and Tb^3+^ (^5^D_4_, 20500 cm^−1^)^7^. And the excitation spectra of **1**·Eu (Figure S4) and **1**·Tb monitored at the strongest f-f' transition at 613 nm and 545 nm resemble the profile of the absorption spectrum. All of these facts demonstrate an energy transfer from the PAnPO_2_ to the lanthanide. The calculated energy transfer rates (*k*_ET_) of **1**·Eu and **1**·Tb, estimated by the equation of *k*_ET_ = 1/*τ* − 1/*τ*_0_^12^,^13^ with **1**·Gd complex as the reference, are beyond 10^8^ s^−1^, indicating an efficient energy transfer from PAnPO_2_ to the lanthanide acceptors. And the Dexter energy transfer mechanism is operative in **1**·Eu and **1**·Tb based on the Calculations of donor/acceptor spectrosocpic overlap with PhotoChemCAD version 2.1^13^.

### Processivity studies on forward and backward walking

As depicted in Figure 2, by increasing its solution concentrations from 1 × 10^−6^ M to 1 × 10^−3^ M (the maximum limited concentration is about 10^−3^ M), the distances between the maximum emission bands of the walker (PAnPO_2_) and those of the stationary (Eu^III^-based subunit) shorten from 146 to 98 nm by ca. 48 nm. When the concentration reaches its highest value in the solid state, the distance remains only 28 nm eventually. These phenomena can be simply viewed as the walker approaches the stationary gradually with concentrations increasing, shielding almost the stationary in the solid state. We have verified that these phenomena are reversible, diluting the solution concentrations from 1 × 10^−3^ M to 1 × 10^−6^ M, the walker leaves the stationary from 98 nm to 146 nm. Due to the non-destructive physical cycle, these phenomena are completely reversible and reliable.

**Figure 3:**
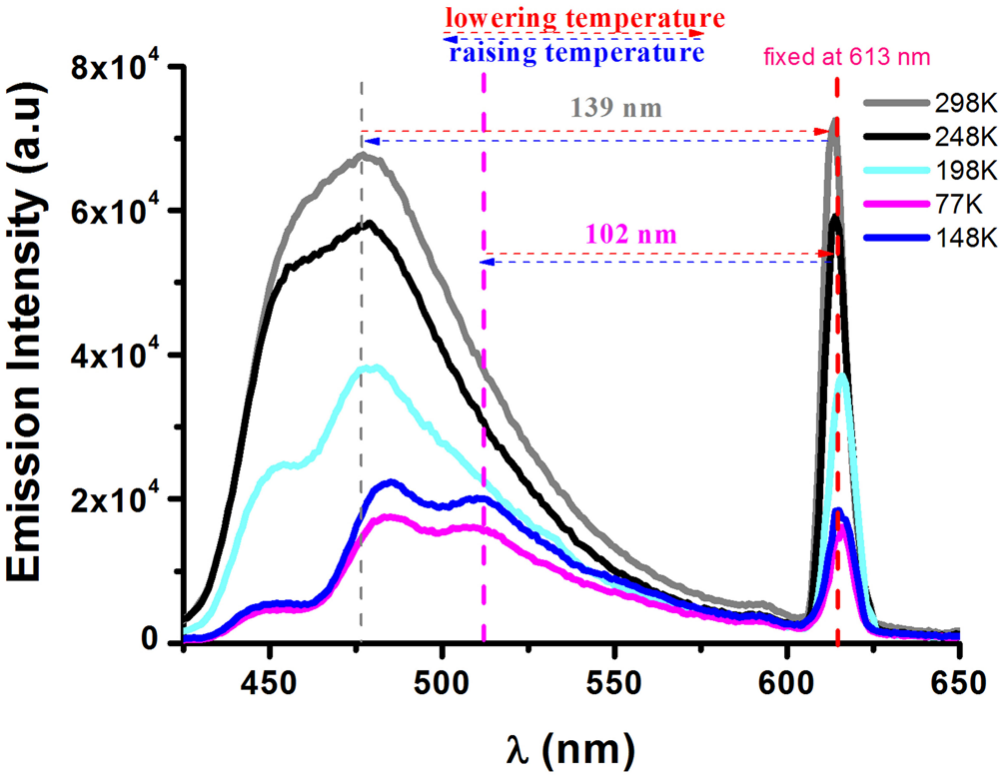
Emission spectra of **1**·Eu (*λ*_ex_ = 350 nm) with the concentration of 1.5 × 10^−5^ M in dichloromethane solution at different temperatures.

Since cooling can also fortify the restriction of the intramolecular breathing vibration process of the walkers, we thus studied the temperature effect on the emission behaviors of **1**·Eu. As shown in Figure 3, by successively decreasing the measurement temperature, the walker approaches the stationary gradually. When the dilute solution of **1**·Eu (1.5 × 10^−5^ M) is cooled to 198 K, the emission peak (*λ*_max_ = 474 nm) of the walker is divided into two vibration peaks (*λ*_max_ = 450 and 478 nm) with a frequency of ca. 1300 cm^−1^. Further dropping the temperature to 148 K, the two vibration peaks are splitted into three vibration ones (*λ*_max_ = 450, 485 and 511 nm) with the vibronic spacings ca. 1600 and 1050 cm^−1^, compared to those of the free PAnPO_2_ (Figure S6, two vibronic shoulders with *λ*_max_ = 450 and 475 nm with a frequency of ca. 1170 cm^−1^), assigned as the low energy ^1^*π* → π* transitions in the anthracenyl ring^11^. Noticeably, the third vibration peak of the walker at 77 K approaches the stationary closer ca. 37 nm (139–102) than the distance between the walker and the stationary at 298 K. Warming up the solution from 77 K to 298 K, the single emission band recovers from three vibration peaks with the distance from the stationary ca. 139 nm.

Interestingly, by increasing the concentration of **1**·Eu up to 5 × 10^−4^ M, the emission spectra and profiles (Figure S7, ESI) of the walker are different from those in diluted solution (1.5 × 10^−5^ M). On one hand, two vibration peaks (*λ*_max_ = 450 and 488 nm) with the vibronic spacing of ca. 1730 cm^−1^ appear at room temperature, which could only be observed until the temperature is cooled to 198 K in diluted solution. Similarly, the second vibration peak (*λ*_max_ = 488 nm) exhibits a larger red-shift than those in diluted solution (*λ*_max_ = 475 nm). On the other hand, instead of three vibration peaks observed in diluted solution, a broad emission band from 480 nm to 600 nm is observed in concentrated solution at 77 K.

Comparison with the emission behaviors of **1**·Eu in dilute (1.5 × 10^−5^ M) and concentrated solution (5 × 10^−4^ M) under 77 K (Figure S8, ESI), we find that not only the emission profiles of the walker change from three vibration peaks (*λ*_max_ = 450, 485 and 511 nm) to a broad emission band (*λ*_max_ = 511 nm), but also the walker approaches the stationary from 163 to 102 nm (ca. 61 nm). This value is larger than that at room temperature (ca. 48 nm), indicating low temperature (77 K, ca. 61 nm) with more effect than high temperature (298 K, ca. 48 nm) on the walking distance.

Although similar to dimers, two bridging parallel anthracenyl rings in **1**·Eu experience a strong *π*···*π* stacking interactions (Figure 1), different emission behaviors of excimers with red-shift and the absence of vibronic structures^8^,^9^, forming by aromatic conjugate molecules in aggregation, are found. Such as, the emission bands of the PAnPO_2_ in **1**·Eu exhibit red shift with vibronic structures (*λ*_max_ = 450, 485 and 511 nm) in diluted solution (1.5 × 10^−5^ M), but without vibronic structures (a broad emission band centred at ca. 510 nm) in concentrated solution (5 × 10^−4^ M) at 77 K. And the vibronic structures (*λ*_max_ = 450 and 488 nm) in concentrated solution (5 × 10^−4^ M) rather than in diluted solution (1.5 × 10^−5^ M) (*λ*_max_ = 474 nm) are observed at 298 K. Therefore, the vibronic structures of **1**·Eu appear to be dependent on the measurement concentrations and temperatures.

**Figure 4:**
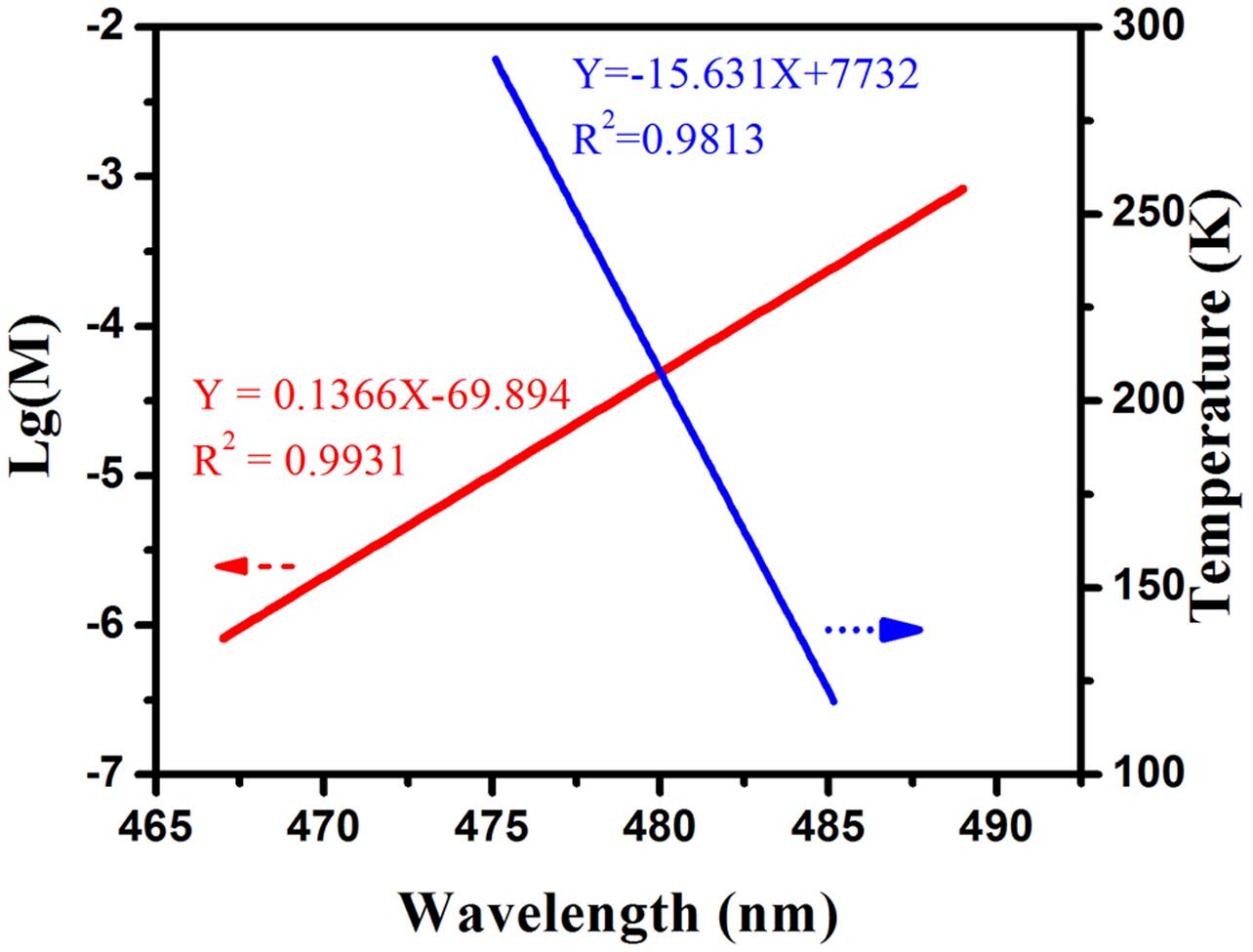
Linear relationships between the maximum emission bands of the walker in **1**·Eu *vs* the concentrations {lg(*M*)} within 10^−3^ M (red line), and the temperatures (*K*) within the range from 120 K to 300 K in 10^−5^ M (blue line).

Taking advantage of such unique properties that the measurement temperatures and concentrations have significant effect on the walking distance of the walker, such materials displays some potential applications on concentration or temperature sensors. Pleasantly, there are good linear relationships between the maximum emission bands of the walker *vs* the concentrations {lg(*M*)} and the measurement temperatures based on some limitations. As shown in Figure 4 and Figures S9–S10 (ESI), we could find that the linear relationships between the maximum emission bands of the walker *vs* the concentrations {lg(*M*)} (*R*^2^ = 0.99) is slightly better than it *vs* the measurement temperatures (*R*^2^ = 0.98).

## Discussion

**Figure 5:**
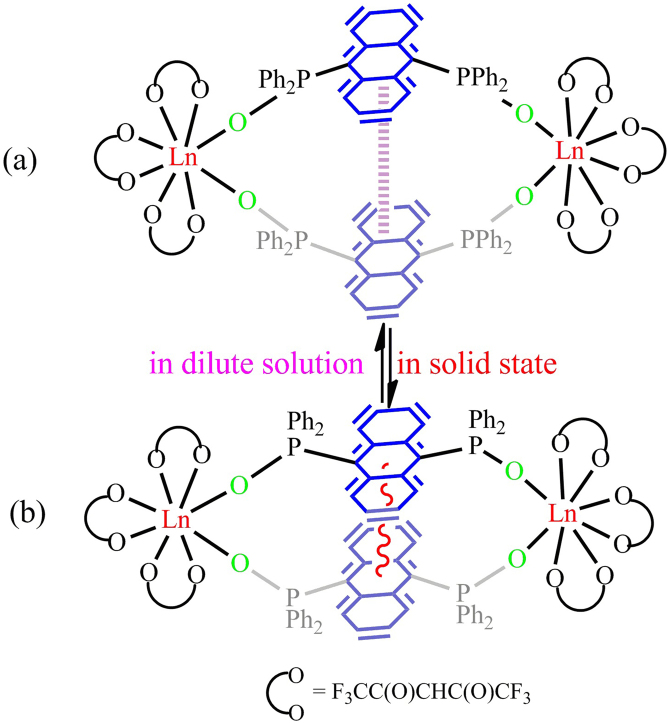
Configuration changes of **1**·Eu in solution and the solid state.

As the measurement temperatures and concentrations have critical effect on the walking distance of the walker in **1**·Eu, it can be concluded that it is the aggregation effect induces such walkable dual emissions. As shown in Figure 5, **1**·Eu displays (a) configuration with a long distance between the two bridging anthracenyl rings in isolated state. However, in the aggregated state, the separation becomes shorter and shorter, due to the *π*···*π* stacking interactions (Figure 5(b)). Crystallographic evidence does confirm a short intramolecular *π*···*π* separation between the two bridging coplanar anthracenyl rings of 3.4 Å. In addition, various emission behaviors of the temperature-dependent vibronic structure also indirectly support the above hypothesis.

Based on the above possible mechanism, asides from acting as sensors of concentration and temperature, such kind of cyclic dimeric lanthanide may be promising in other relative applications^18^,^19^, such as in information gateway or molecular electronics^19^, by elaborately modulating the distance between the two bridging anthracenyl rings.

**Figure 6:**
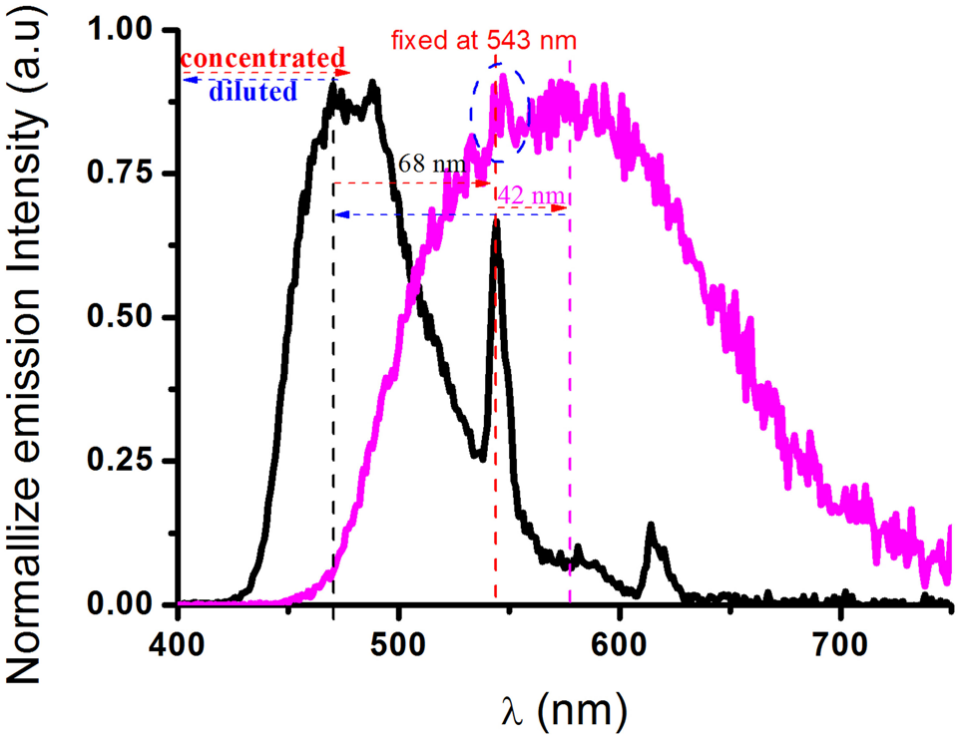
Normalized emission spectra of **1**·Tb (*λ*_ex_ = 330 nm) in dichloromethane solution (1.5 × 10^−5^ M, black) and the solid state (red) at ambient atmosphere, showing the emission bands of the walker (PAnPO_2_) across those of the stationary (Tb^III^-emitter).

In order to realize the walker crossing the stationary, two approaches may be practicable. One is blue-shifting the emission bands of the stationary, such as using Tb(hfac)_3_ instead of Eu(hfac)_3_ to fix the central emission band of the stationary from 613 nm to 543 nm. The other one is using more conjugate luminophores than PAnPO_2_ as the walker. As expected, with the former approach, we have realized the crossing dual emission bands in **1**·Tb. As shown in Figure 6, **1**·Tb exhibits dual emissions at ca. 475 nm and 543 nm in dilute solution (1.5 × 10^−5^ M) from both the walker (PAnPO_2_) and the stationary (Tb^III^-centred emitters), respectively. When the concentration reaches its highest value in the solid state, the walker crosses the stationary (543 nm) with a beyond distance of 42 nm. Similarly, under dilute conditions, the walker moves backward about 110 nm (68 + 42) to recover its initial state. These phenomena could thus be simply viewed as: there is a small island in a gulf, when the tide rises, seawater climbs across and drowns the island; once the tide ebbs away, the island would stand out again.

Noteworthily, it is the first case that emission bands from the sensitizer could reversibly cross or leave the fixed Ln^III^-based emissions depending on temperature and concentration, although dual emissions arising from both the sensitizers and lanthanide ions always could be observed in a hybrid lanthanide due to a part energy transfer^15^,^16^. On the other hand, if other metallic blocks complexed with the same ligand, dual emissions may also be provided^20^,^21^. However, as the energy levels of metallic blocks and the aromatic ligands are susceptible to the external environment, the two emissions are active during altering experimental conditions^22^.

In summary, walkable dual emissions in the cyclic **1** have been found, and the emission bands of the walker are able to pass through and reverse back those of the stationary depending on temperature and concentration. Due to the non-destructive physical cycle, these phenomena are verified to be completely reversible and reliable. This work establishes a model that the information between the walker and the stationary could be exchanged and dispatched by the “WDE”, through switching the intramolecular interactions in dimeric complexes. Additionally, our results demonstrated that such materials are promising for potential application as sensors of concentration and temperature.

## Methods

### Sample preparation

All manipulations were performed under dry argon atmosphere using Schlenk techniques and a vacuum-line system. The solvents were dried, distilled, and degassed prior to use, except those for spectroscopic measurements were of spectroscopic grade. Hexafluoroacetylacetone (Hhfac) were commercially available. Ln(hfac)_3_(H_2_O)_2_ (Ln = Eu, Gd, Tb)^23^, and 9,10-bis(diphenylphosphino)anthracene (PAnP)^11^ were prepared by the literature procedures.

### Synthesis of PAnPO_2_

A mixture of diphosphine (1040 mg for PAnP), hydrogen peroxide (0.5 mL, 30%), and absolute ethanol (40 ml) was refluxed for 30 min. The solution was poured into water (ca. 100 mL) and extracted with chloroform. The organic extract was dried over magnesium sulfate, filtered, and concentrated to afford diphosphine dioxides powder as a yellowish green solid, ESI-MS (CH_3_OH-CH_2_Cl_2_, m/z): 579 (100) [M + H]^+^, mp: 264–265°C.

### General procedures for the preparation of {Ln(hfac)_3_PAnPO_2_}_2_, (1, Ln = Eu, Tb)

#### Method 1

Reaction Ln(hfac)_3_(H_2_O)_2_ (Ln = Eu, Gd, Tb) (0.1 mmol) with PAnPO_2_ in 30 mL dichloromethane at room temperature until the solution became clear. After filtered, crystallization by layering *n*-hexane onto the corresponding concentrated dichloromethane solutions afforded yellow crystals.

#### Method 2

Ln(hfac)_3_(H_2_O)_2_ (Ln = Eu, Gd, Tb) (0.1 mmol) and equal molar ratio of PAnP were stirred in 30 mL dichloromethane at ambient atmosphere until the solution became clear. After filtered, crystallization by layering *n*-hexane onto the corresponding concentrated dichloromethane solutions afforded the products as crystals.

**1**·Eu. Anal. Calcd for C_106_H_66_Eu_2_F_42_O_16_P_4_·H_2_O: C, 44.79; H, 2.41. Found: C, 44.74; H, 2.41. ESI-MS (CH_3_OH–CH_2_Cl_2_): m/z (%) 1352 [M/2]. IR (KBr, cm^−1^): 1650s (C = O). Yield: 78%.

**1**·Gd. Anal. Calcd for C_106_H_66_Gd_2_F_42_O_16_P_4_·H_2_O: C, 44.63; H, 2.40. Found: C, 44.64; H, 2.42. IR (KBr, cm^−1^): 1651s (C = O). Yield: 81%.

**1**·Tb. Anal. Calcd for C_106_H_66_Tb_2_F_42_O_16_P_4_·H_2_O: C, 44.60; H, 2.40. Found: C, 44.54; H, 2.37. IR (KBr, cm^−1^): 1651s (C = O). Yield: 75%.

### Physical measurements

Elemental analyses (C, H, N) were carried out on a Perkin-Elmer model 240C elemental analyzer. Electrospray ion mass spectra (ESI–MS) were performed on a Finnigan LCQ mass spectrometer using dichloromethane-methanol mixture as mobile phases. UV-vis absorption spectra were measured on a Perkin-Elmer Lambda 35 UV-vis spectrophotometer. Infrared (IR) spectra were recorded on a Magna750 FT-IR spectrophotometer with KBr pellet. Emission, excitation spectra and emission lifetimes were recorded on an Edinburgh Instrument (FLS 920 spectrometer). Low temperature emission and excitation spectra were carried out in an oxford instrument liquid nitrogen cryostat, Optistat^DN^. The instrument response function at the excitation wavelength was deconvolved from the luminescence decay. The quantum yields of **1**·Eu with white-light emission was determined relative to that of [Ru(bpy)_3_]Cl_2_ (*Φ*_em_ = 0.028) in H_2_O. All the quantum yields were calculated by *Φ*_s_ = *Φ*_r_(*A*_r_/*A*_s_)(*I*_r_/*I*_s_)(*n*_s_/*n*_r_)^2^(*D*_s_/*D*_r_)^24^, where the subscripts r and s denote reference standard and the sample solution, respectively; and *A*, *n*, *I*, *D* and *Φ* are the absorbance of a sample at excitation wavelength *λ*, the refractive index of the solvents, the relative intensity of excitation light at wavelength *λ*, the integrated intensity and the luminescence quantum yield, respectively. All the solutions used for determination of emission lifetimes and quantum yields were prepared under vacuum in a 10 cm round bottom flask equipped with a side arm 1 cm fluorescence cuvette and sealed from the atmosphere by a quick-release teflon stopper. Solutions used for luminescence determination were prepared after rigorous removal of oxygen by three successive freeze-pump-thaw cycles. Calculations of the chromaticity coordinates (CIE) of **1**·Eu with ColorCoordinate. exe version 1.0 was carried out with the concentration of 1.5 × 10^−5^ M in dichloromethane solutions under irradiation with 350 nm in dichloromethane at ambient temperature.

### Crystal structural determination

Single crystal of **1**·Eu·H_2_O suitable for X-ray diffraction was grown by layering *n*-hexane onto the corresponding dichloromethane solutions. Crystals coated with epoxy resin or sealed in capillaries with mother liquors were measured on a SIEMENS SMART CCD diffractometer by *ω* scan technique at room temperature using graphite-monochromated Mo-Kα radiation (*λ* = 0.71073 Å). Lp corrections were carried out in the reflection reduction process. The structures were solved by direct method and the heavy atoms were located from E-map. The remaining non-hydrogen atoms were determined from the successive difference Fourier syntheses. The non-hydrogen atoms were refined anisotropically except for the F atoms, and the hydrogen atoms were generated geometrically with isotropic thermal parameters. The structures were refined on *F*^2^ by full-matrix least-squares methods using the SHELXTL-97 program package^25^, the refinements were carried out by fixing the C–F distances (1.32 ± 0.01 Å) with the occupancy factors of F1–F36 and F1′–F36′ being 0.50, respectively. Crystallographic data of **1**·Eu·H_2_O was summarized in Table S2 (ESI).

## Author Contributions

H.X. and P.J. carried out the experimental work. All the authors contributed to the design of the experiments, the analysis of the data, and H.X. finished the writing of the paper.

## Supplementary Material

Supplementary Information Supplementary Information
