# Supplementary material for: Walkable Dual Emissions
Source: Sci Rep. 2013 Jul 16;3:2199. doi: 10.1038/srep02199 (PMC3712315; doi:10.1038/srep02199)
Supplement: Supplementary Information [file srep02199-s1.doc]

**Supplementary Information**

**Walkable Dual Emissions**

Hai-Bing Xu,[[1]](#footnote-2)*a,b Peng-chong Jiao,^a^ Bin Kang,^a^ Jian-Guo Deng,^a^ and Yan Zhang^a^

*^a^ New Materials R&D Center, Institute of Chemical Materials, China Academy of Engineering Physics, Chengdu, 621900, China*

*^b^ State Key Laboratory of Structural Chemistry, Fujian Institute of Research on the Structure of Matter, Chinese Academy of Sciences, Fuzhou, Fujian 350002, China*

Table S1. Emission Data of Compounds at 298 K

| Compounds^[a]^ | Medium | *λ*_em_/nm (*τ*_em_) |
| --- | --- | --- |
| **PAnPO_2_** | Solid | 490 (3.6 ns) |
|  | CH_2_Cl_2_ | 470 (13 ns) |
| **1⋅**Eu | Solid | 585 (1.5 ns) |
|  | CH_2_Cl_2_ | 475 (8.8 ns) |
|  |  | 613 (238 us) |
| **1⋅**Gd | CH_3_OH | 477 (9.7 ns) |
| **1⋅**Tb | Solid | 585 (1.6 ns) |
|  | CH_2_Cl_2_ | 475 (9.4 ns) |
|  |  | 543 (19.5 us) |

[a] Excitation wavelength in the lifetime measurement is 384 nm for **PAnPO_2_**, 350 nm for **1⋅**Eu, 330 nm for **1⋅**Tb, and 380 nm for **1⋅**Gd with the concentrations of 1.5×10^-5^ M.

Table S2. Crystallographic Data of 1⋅Eu·H_2_O

|  | **1⋅**Eu·H_2_O |
| --- | --- |
| empirical formula | C_106_H_64_Eu_2_F_36_O_17_P_4_ |
| fw | 2721.37 |
| space group | *C*2/*c* |
| *a*, Å | 29.558(8) |
| *b*, Å | 22.879(5) |
| *c*, Å | 20.198(6) |
| *α*, ° | 90.00 |
| *β*, ° | 123.273(3) |
| *γ*, ° | 90.00 |
| *V*, Å^3^ | 11420(5) |
| *Z* | 4 |
| *ρ*_calcd_ g/cm^-3^ | 1.583 |
| *μ*, mm^−1^ | 1.265 |
| Radiation (*λ*, Å) | 0.71073 |
| temp, (K) | 293(2) |
| *R*1(*F*_o_)^a^ | 0.0595 |
| *wR*2(*F*_o_^2^)^b^ | 0.1491 |
| GOF | 1.200 |

^a^ *R*1 = Σ*F*_o_ - *F*_c_/Σ*F*_o_ ^b^*wR*2 = Σ[w(*F*_o_^2^ – *F*_c_^2^)^2^]/Σ[w(*F*_o_^2^)]^1/2^


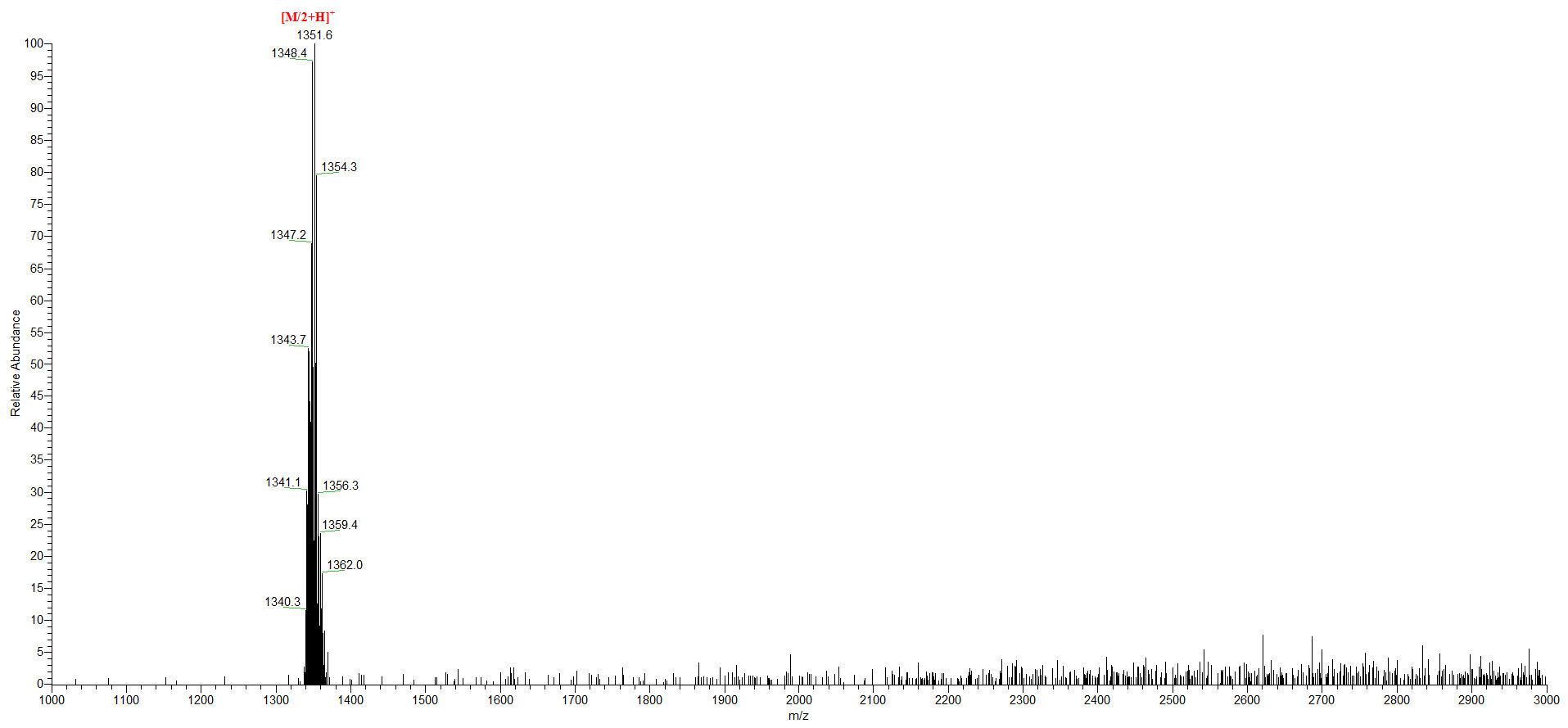


**Figure S1.** Positive ion ESI-MS of **1**⋅Eu

**Figure S2**. UV-vis absorption spectra of **1**⋅Eu (red) and the free PAnPO_2_ (black) in dichloromethane solutions at ambient temperature.

**Figure S3**. Emission spectra of **1**⋅Gd (*λ*_ex_= 380 nm) with the concentration of 1.5×10^-5^ M in methanol solutions at 77K.

**Figure S4**. Absorption (short dash) and excitation spectra (solid) with *λ*_em_ = 613 nm (solid line) of **1**⋅Eu. The excitation spectrum is corrected for lamp response.


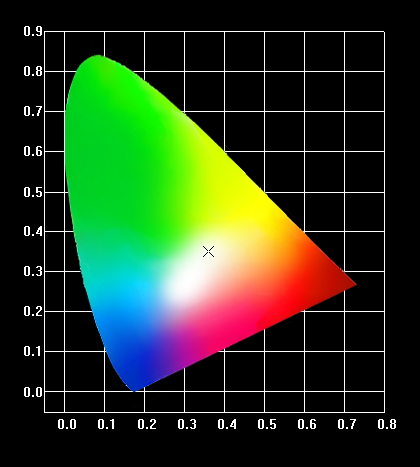


**Figure S5**. The chromaticity coordinates (0.36, 0.35) of **1**⋅Eu with the concentration of 1.5×10^-5^ M in dichloromethane solutions under irradiation with 350 nm in dichloromethane at ambient temperature

**Figure S6**. Emission spectra of the free PAnPO_2_ (*λ*_ex_= 384 nm) with the concentration of 1.5×10^-5^ M in dichloromethane solutions at different temperature.

**Figure S7.** Emission spectra of **1**⋅Eu (*λ*_ex_= 350 nm) with the concentration of 5×10^-4^ M in dichloromethane solution at different temperatures.

**Figure S8**. Normalized emission spectra of **1**⋅Eu (*λ*_ex_= 350 nm) with the concentration of 5×10^-4^ M (red) and 1.5×10^-5^ M (blue), and the free PAnPO_2_ (*λ*_ex_= 384 nm) at 1.5×10^-5^ M (black) in solutions at 77 K.

**Figure S9**. Normalized Emission spectra of **1**⋅Eu (*λ*_ex_ = 350 nm) with increasing concentrations in dichloromethane solutions at ambient atmosphere, showing the emission bands of the walker (PAnPO_2_) toward that of the stationary (Eu^III^-emitter).

**Figure S10**. Normalized emission spectra of **1**⋅Eu (*λ*_ex_ = 350 nm) with the concentration of 1.5×10^-5^ M in dichloromethane solutions at different temperature, showing the emission bands of the walker (PAnPO_2_) toward that of the stationary (Eu^III^-emitter).

1. ^^ To whom correspondence should be addressed. E-mail: hbxu0513@yahoo.com.cn [↑](#footnote-ref-2)
